# The use of a dual mobility cup in the management of recurrent dislocations of hip hemiarthroplasty

**DOI:** 10.1007/s10195-015-0365-8

**Published:** 2015-07-18

**Authors:** Christian Carulli, Armando Macera, Fabrizio Matassi, Roberto Civinini, Massimo Innocenti

**Affiliations:** Orthopaedic Clinic, University of Florence, Largo P. Palagi 1, 50139 Florence, Italy

**Keywords:** Dislocation, Hemiarthroplasty of the hip, Dual mobility cups, Revision

## Abstract

**Background:**

Dislocation is one of the most frequent causes of failure of hemiarthroplasties of the hip, which is the most common treatment for femoral neck fractures in elderly patients. A revision with conversion to total hip arthroplasty is the gold standard in case of failure of closed reduction: however, the use of standard or modular components shows variable outcomes. The use of a dual mobility cup has been evaluated in patients with unstable implants, given the good outcomes obtained in primary and revision surgery. The aim of this study was to assess the results of revisions by dual mobility cups in unstable hemiarthroplasties.

**Materials and methods:**

Thirty-one patients (mean age 75.4 years) were retrospectively evaluated between 2006 and 2010 after conversion to total hip arthroplasty with dual mobility cups for recurrent dislocations. The mean number of dislocations was 2.6 (range 2–5). The evaluation was performed by the American Society of Anesthesiologists physical function score (ASA) and the Harris hip score, and several radiologic criteria.

**Results:**

The mean follow-up was 3.8 years. No recurrence of dislocation was recorded. The ASA score remained unchanged, and the mean Harris hip score improved from 62.2 before dislocation to 76.0 points postoperatively.

**Conclusions:**

Dual mobility cups may be a useful option in the treatment of a hemiarthroplasty dislocation. No risk of a new revision due to instability after insertion of dual mobility cups resulted in our experience, and this option may be strongly considered in cases of revisions of unstable hemiarthroplasties.

*Level of evidence* IV.

## Introduction

Dislocation is one of the major causes of failure of a hemiarthroplasty of the hip (HAH). Its incidence is rated at 6–10 % with respect to 2–3 % for total hip arthroplasty (THA) [1, 2]. Dislocations occur typically within 6 months after surgery [3], particularly in the first 2–6 weeks. Several factors have been advocated, such as sex, cognitive status, anatomy of the acetabulum (related to patients); femoral head diameter, femoral stem rotation and off-set, surgical approach and excessive removal of joint capsule (related to surgeons) [4, 5]. It is crucial to understand the causes of dislocation before facing surgery with an adequate strategy, in order to limit the recurrence of the instability. Several procedures have been proposed depending on the cause of the dislocation: repositioning of femoral stem [6], conversion to THA [6, 7], revision with traditional or modular neck components [7–10], use of constrained components [11, 12], trochanteric advancement [13], removal of acetabular or femoral osteophytes [6], and repair of the abductor muscles and of the joint capsule [14, 15]. However, all these procedures showed rates of success ranging from 60 to 80 %, independently by the cause leading to instability [6, 10, 13, 16–19]. Particularly, the conversion of HAH to THA demonstrated discouraging results with reports of even worse failure rates than a full revision [6, 7]. The implant of constrained acetabular inserts also showed variable results, with a high risk of increased wear, osteolysis, and instability in THA [11, 12]. Revisions of unstable THAs are generally considered technically demanding procedures [20–22]. Recently, good results have been obtained by the use of “dual mobility” cups for revisions of unstable THAs [23–31] and primary implants after femoral neck fractures [32], in terms of limitation of dislocation recurrence and preservation of a wide range of motion (ROM): low wear is also expected. To date, no report addresses similar outcomes for the management of unstable HAHs treated by revisions with dual mobility cups.

The purpose of this study was to assess the short-term results of a series of patients affected by unstable HAHs managed by a conversion to THA with dual mobility cups.

## Materials and methods

**Fig. 1:**
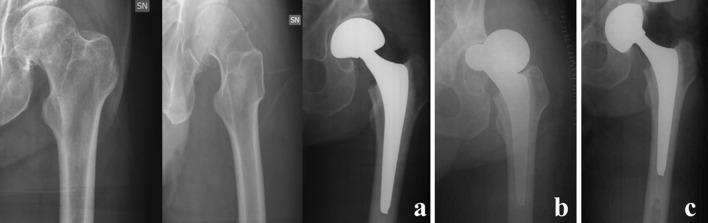
A left femoral fracture of a 72-year-old male patient, treated by a hemiarthroplasty of the hip (**a**); 3 weeks postoperatively, a dislocation of the implant occurred (**b**), and conversion to total hip replacement by a pressfit dual mobility cup was performed (**c**)

**Fig. 2:**
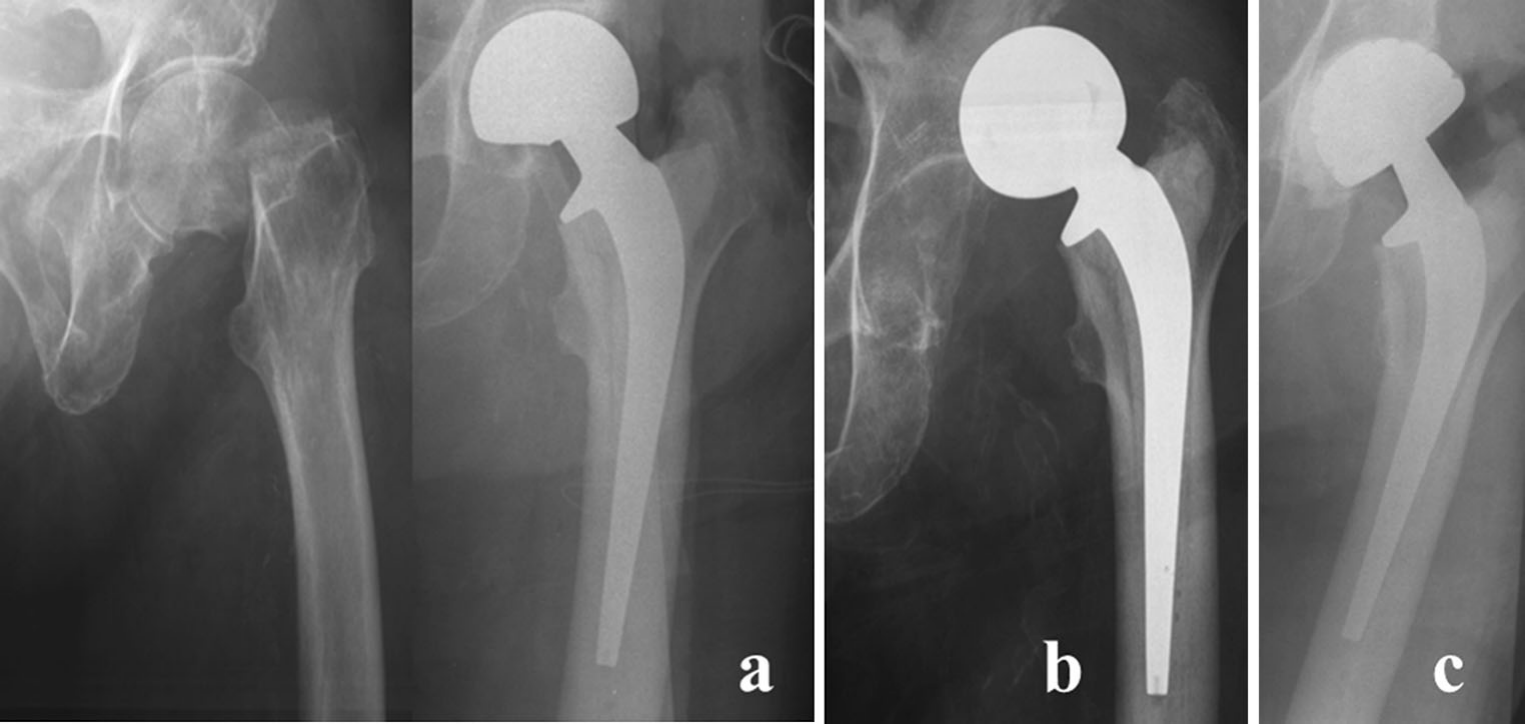
A left femoral fracture of a 79-year-old female patient, affected by Alzheimer’s disease, and treated by a hemiarthroplasty of the hip (**a**); 4 days after surgery, a dislocation occurred, treated by closed reduction under anaesthesia (**b**). A second dislocation recurred after 5 days, thus a cemented dual mobility cup was implanted (**c**)

We retrospectively reviewed 31 patients (31 hips) affected by recurrent dislocations of HAH, treated by a conversion to THA with dual mobility cups between 2006 and 2010. All patients had been given bipolar cemented implants for femoral neck fractures: the index operation was performed with a mean interval of 2.4 days (range 1–3) after patient admission to the emergency room. Eighteen patients were female and 13 male, with a mean age of 75.4 years (range 71–86) at the time of fracture. The right side was affected in 17 cases; the left side in 14 cases. Eleven patients were operated on in other hospitals, while 20 were operated on at the authors’ institution. All patients were operated on by a lateral approach at the time of HAH. The mean interval to the first dislocation after HAH was 23.2 days (range 1–46). The mean number of dislocations was 2.6 (range 2–5). Dislocations were mostly posterior (29 cases); one subject showed a dislocation in an anterior direction; only one case was multidirectional (a single patient with five episodes of instability).An evaluation of the associated risk factors of patients was made before proceeding to revision. The mean time between the HAH and the revision in arthroplasty was 3.2 years (range 7 months–6 years). The American Society of Anesthesiologists physical function (ASA) score based on the severity of patients’ comorbidities was evaluated [33]. The ASA score at the time of revision was III in 19 patients, IV in six subjects, and II in the remainder. Several pathologies were present, and a high risk of dislocation was considered in some patients: three cases of Parkinsonʼs disease, three cases of diabetes mellitus with severe peripheral neuropathy, one case of critical peripheral arterial disease, two severe cognitive impairments related to Alzheimerʼs disease, one hemiparesis as the result of a previous stroke, and one of severe pluriarticular rheumatoid arthritis. The Harris hip score (HHS) was also recorded [34]. A radiographic study by anteroposterior and lateral views was conducted to study the femoral stem position according to Loudon and Charnley [35], and the stability of the components as described by Engh et al. [36]. The presence of radiolucent lines and osteolysis of periprosthetic bone were assessed by the criteria of DeLee and Charnley, and Gruen et al. [37, 38]. Cup inclination was assessed in the anterior–posterior projection, measuring in degrees the angle formed by a line drawn along the bottom of the acetabular component intersecting with the horizontal inter-teardrop line. Hip centre restoration was assessed by calculating the perpendicular distance from the prosthetic centre of rotation to a horizontal line drawn between the tips of the teardrops. Limb length was evaluated. Finally, the presence of periarticular ossification was also evaluated by Brooker’s classification [39]. Collaborative patients, or relatives of poorly oriented subjects were adequately informed, and approved the treatment and follow-up. Surgery was performed by two surgeons, in all cases by a direct lateral approach through the previous surgical scars. In 19 cases a general anaesthesia was performed (ASA score: IV in six patients, III in 13); in 12 cases, a locoregional anaesthesia was chosen. In 25 cases, a capsular laxity was present, while in the remaining patients the capsule was mostly absent. When possible, capsulae were sutured and soft tissues reconstructed after the cup positioning. In all cases a dual mobility acetabular cup was implanted as porous coated press-fit or cemented (Avantage^®^, Biomet, Warsaw, IN, USA). This component consisted of a metal cup with a polished inner surface articulating with a high molecular weight polyethylene bipolar insert (acting as a large diameter head) containing a 28-mm chrome–cobalt head. In 20 cases, a press-fit cup was implanted (Fig. 1): three cups needed a further fixation by two or three acetabular screws. In the remainder, a cemented cup was implanted (Fig. 2). Criteria leading to the use of a cemented cup were poor bone quality or a significant enlargement of the native diameter of the acetabulum as tested intraoperatively during acetabular preparation. Cups sizes between 44 and 56 mm were used. Actually, in a single case we also proceeded to the revision of the cemented femoral stem, given the remarkable rotational malposition of the component and the length discrepancy (2 cm): a new larger cemented femoral stem was used (MS-30^®^, Biomet, Warsaw, IN, USA). In 12 patients, a long (eight cases) or extra-long (four cases) 28-mm head was implanted to ensure an adequate offset and further stability. The prophylaxis of heterotopic ossifications was made by Indometacin 25 mg t.i.d. for 3 weeks in patients without any contraindications related to other comorbidities or concomitant therapies. Parameters such as blood loss, following the criteria of Liu et al. [40], surgical time, and early postoperative complications were recorded. Postoperative care consisted of a short period of immobilization with a pillow between the legs in order to limit adduction of the hips. An assisted passive motion protocol from the 3rd postoperative day was then performed. Active exercises, partial weight-bearing, and assisted gait activities were then specifically prescribed for each case, depending on pain and patients’ collaboration. All patients were clinically and radiographically evaluated at 1 month after surgery, and after 3, 6, and 12 months. After this follow-up, all the subjects were encouraged to attend a yearly follow-up.

Considering the small size of the study population, only the Wilcoxon signed rank test was used to compare pre- and postoperative HHS scores.

## Results

All patients were followed at least for 2 years, with a mean follow-up of 3.8 years (range 2–7 years). The average blood loss was 210 cc (range 100–400), and the mean surgical time was 57.8 min (range 45–120). Seven patients were assisted after surgery in an intensive care unit for 24–48 h. No intraoperative complication was recorded. Postoperative complications were present in six cases (19.3 %): three deep vein thromboses (one unilateral, one bilateral) managed by a mechanical compression and therapeutic doses of low-molecular-weight heparin; one case of urinary tract infection, treated by antibiotics; one case of superficial wound infection, managed by an advanced wound care treatment and oral antibiotics; and one case of an acute imbalance in diabetes mellitus, managed by tailored insulin therapy.

No case of dislocation was recorded during the mentioned follow-up. Radiographic studies revealed radiolucent lines in zone 2 according to DeLee and Charnley in three patients (all with cementless cups). However, these were not progressive and were less than 2 mm in width: these cups were correctly implanted. In three additional cases radiolucent lines of about 1 mm without progression around the femoral component were found in zone 1 (the only patient with the stem revision) and zone 5 (two patients) according to Gruen et al. The mean cup inclination was 45.4° (range 42–49°). An adequate hip centre restoration was achieved in 23 cases. A suboptimal hip centre was achieved in the remaining subjects; however, due to good stability, the patients accepted well the residual length discrepancy (in all cases <1.5 cm). No osteolysis, significant subsidence, or cement mantle fractures were noted, according to the criteria of Loudon and Charnley. No implant was found to be unstable or poorly stable according to Engh’s classification. We recorded three cases (9.6 %) of heterotopic ossifications grade 1 and one grade 2 (the patient with the revised stem), without, however, referred symptoms or functional impairments: two of them did not undergo prophylaxis due to clinical contraindications.

The pillow was maintained for an average interval of 2.8 days (range 2–4). The mean HHS improved from 62.2 points (range 34–75) before the dislocation to 76.0 points (range 71–80) postoperatively with a significant difference (*p* = 0.002). The ASA score remained basically stable after surgery in all the patients. Symptoms and functional disability progressively decreased over the follow-up period, allowing all patients without neurologic impairments to return to their common daily activities. Poorly or uncollaborative patients were not substantially able to complete a full functional recovery, however, without further episodes of dislocation.

## Discussion

Dislocations of HAHs are generally associated with an insufficient restoration of the centre of rotation or other mechanical problems due to a wrong primary implantation. The conversion of an unstable HAH to a standard THA is a procedure with a high risk of further dislocations, with an incidence often higher than revision THA itself [2, 20–22, 41, 42]. Several reasons have been advocated: the reduction of the diameter and offset of the femoral head, which may produce an inadequate soft tissues tension; the inappropriate positioning of a retained femoral stem, frequently maintained to avoid long surgical procedures in critical patients; and the insufficient retaining properties of the acetabular cup/liner complex. Several other options such as the use of a cemented cup with a structural bone graft fixed with screws, threaded cups with or without bone grafting, constrained cups, reinforcement rings, or “anti-protrusio” cages have been proposed over the decades. Variable results have been obtained in cases of acetabular discontinuity or severe bone loss, poor acetabular rim coverage, and substantial alterations of shape of the acetabulum [43, 44]. In the remaining cases, outcomes were not satisfactory.

Figved et al. [20] reported a lower risk of complications, including instability, based on the Norwegian Arthroplasty Register, in cases of conversion of HAH to THA with stem revisions, compared to stem retaining procedures. Moreover, in the same series, modular implants for revision presented more advantages related to head size, neck length, and worn head replacement. However, no mention of dual mobility cups has been described.

Only a few studies showed no relationships or even higher rates of dislocation between large diameter heads and the risk of instability in primary and revision implants [41, 42]. Llinas et al. [21] reported the long-term outcomes of a series of failed HAHs treated with THA with traditional components: higher rates of earlier radiologically detected loosening of acetabular components inserted following HAH failure were found with respect to primary THAs. No mention of dual mobility cups was made in this series.

Constrained cups and liners have been proposed over the years with variable results [11, 12]. Reduction of ROM related to component impingement, increased wear related to high local stresses, and higher risk of loosening were considered the reasons related to significant rates of failure of these implants [23–25].

Dual mobility cups and large femoral heads have their rationale in limiting instability, ensuring a wide ROM with respect to traditional implants, and maintaining low wear in primary and revision hip arthroplasties. Satisfactory long-term outcomes have been reported in several series in primary and revision hip arthroplasty [23–31, 45]. A single multicentre study reported the use of this type of implant for the primary replacement in patients affected by a femoral fracture: a dislocation occurred in three cases out of 214 patients (1.4 %) within the first 3 months [46]. The authors found no recurrence of the dislocation in these patients treated by closed reduction under general anaesthesia, even if they used a posterior approach, generally associated with a higher risk of dislocation with respect to the direct lateral approach [47, 48]. However, to date there has been no significant experience regarding series of HAHs failed for instability and managed by revision with dual mobility cups. Bouchet et al. reported a statistically lower risk of dislocation for the dual mobility cup compared to a conventional 28-mm head and polyethylene inserts implanted through a posterior approach. The instability rate was 0 % compared with 4.63 % for the conventional prostheses [25]. In our series, we recorded improvements in the HHS, and complication rates were comparable to other reports in the literature. Nonetheless, we had no recurrence of dislocation, and no specific failure related to choice of implants. A specific mechanism of failure of dual mobility cups is effectively represented by the intraprosthetic dislocation [49–51]. It consists of the loss of the polyethylene retentive rim, with escape of the femoral head from the liner that may manifest particularly in younger, high-demand patients undergoing a primary THA with this implant [28, 51]. No similar complication was recorded in our series.

The present study has some limitations. It is a retrospective analysis with a small number of patients, and without a control group. However, we do not usually perform revisions with standard or constrained cups for unstable HAHs, using in most cases a dual mobility component: related costs are similar to other choices of treatments. Nevertheless, at short-term follow-up we had no recurrence of instability, with both versions (cemented and cementless) of the dual mobility cup.

We feel that dual mobility cups may be a useful and effective option worth considering in the treatment of HAH dislocations.
